# Carbon-Based Electrocatalyst Design with Phytic Acid—A Versatile Biomass-Derived Modifier of Functional Materials

**DOI:** 10.3390/ijms231911282

**Published:** 2022-09-24

**Authors:** Magdalena Gwóźdź, Alina Brzęczek-Szafran

**Affiliations:** Faculty of Chemistry, Silesian University of Technology, Krzywoustego 4, 44-100 Gliwice, Poland

**Keywords:** phytic acid, biomass, sustainable carbon material, oxygen reduction reaction (ORR), P-doped carbon, electrocatalysis

## Abstract

Increasing energy demands exacerbated by energy shortages have highlighted the urgency of research on renewable energy technologies. Carbon materials that can be employed as advanced electrodes and catalysts can increase the accessibility of efficient and economical energy conversion and storage solutions based on electrocatalysis. In particular, carbon materials derived from biomass are promising candidates to replace precious-metal-based catalysts, owing to their low cost, anti-corrosion properties, electrochemical durability, and sustainability. For catalytic applications, the rational design and engineering of functional carbon materials in terms of their structure, morphology, and heteroatom doping are crucial. Phytic acid derived from natural, abundant, and renewable resources represents a versatile carbon precursor and modifier that can be introduced to tune the aforementioned properties. This review discusses synthetic strategies for preparing functional carbon materials using phytic acid and explores the influence of this precursor on the resulting materials’ physicochemical characteristics. We also summarize recent strategies that have been applied to improve the oxygen reduction performance of porous carbon materials using phytic acid, thereby offering guidance for the future design of functional, sustainable carbon materials with enhanced catalytic properties.

## 1. Introduction

As energy crises and environmental regulations associated with fossil fuel consumption have intensified, extensive investigations have focused on developing technologies to make clean and sustainable energy more accessible. Significant efforts have been devoted to efficient electrochemical energy conversion and storage processes, which are integral for devices such as fuel cells, batteries, supercapacitors, and solar cells. Research regarding proton-exchange membrane fuel cells (PEMFCs) and rechargeable metal–air batteries—both of which rely on the electrochemical oxygen reduction reaction (ORR)—aims to replace precious metal catalysts with inexpensive sustainable alternatives.

Carbon nanomaterials that exhibit high electrocatalytic activity and anti-corrosion performance and can be manufactured using relatively simple and inexpensive processes are promising alternatives to conventional noble-metal-based catalysts [1]. Recently, the use of biomass for preparing sustainable functional carbon materials has become an attractive alternative to the use of conventional fossil fuels, which are nonrenewable, expensive, and yield toxic byproducts upon carbonization [2]. Various naturally occurring precursors—such as wood [3], chitosan [4], cattle bones [5], folic acid [6], soybean shells [7], prawn shells [8], coconut shells [9], saccharides [10,11,12,13], cellulose [14], lignin [15], spongin scaffolds [16], seaweed [17], and lemon peel [18]—have been used to synthesize carbon materials via chemical, biological, or thermochemical processes [19]. Despite the fact that significant progress in using more environmentally benign precursors has been observed recently, increased attention must be also paid to the synthetic methods, which often lack sustainable character (e.g., involve physical and chemical activation with KOH and ZnCl_2_).

The chemical composition, structure, and form/state (i.e., solid or liquid/liquefiable biomass) of the precursors lead to carbon materials with varying carbon content, morphology, porosity, heteroatom/metal doping, and overall yield. Recent progress in the synthesis of bio-derived carbon materials has contributed to the development of products with electrocatalytic activity comparable to that of commercial noble metal catalysts [20].

Environmental and economic issues have highlighted the need for carbon materials that can be fabricated using simple synthetic procedures and inexpensive, abundant precursors that ideally provide carbon materials with different specific functionalities at once, acting as “all-in-one” precursors. Naturally occurring phytic acid (PA) (also known as inositol hexakisphosphate, or IP6) is a precursor that can be introduced at different stages of the fabrication process. Following carbonization, PA can produce carbon materials with a high carbonization yield, high specific surface area, and desirable phosphorous doping [21]. When used as a modifier, PA can alter the properties of existing carbon (nano)materials in terms of their morphology, porosity, and heteroatom doping [22]. Moreover, it can promote the uniform distribution of metals by limiting their aggregation [23].

PA is present in various cereals, legumes, nuts, oilseeds, and tubers (its content ranges from 0.1% to 9.4%) [24]; thus, it is commonly extracted with aqueous acids [25,26]. Owing to its presence in common plant foods, the nutrient properties of PA have been studied since 1985 [27]. It has been established that PA is a natural antioxidant [28,29] that regulates the metabolic processes of many plants [30] and can chelate multivalent cations [30] and proteins [31]. In addition, PA–metal complexes are insoluble in water and stable (i.e., resistant to degradation) at temperatures up to 120 °C [32]. Several review articles have discussed the antioxidant, anti-carcinogenic, and antidiabetic properties of PA [33,34,35,36].

**Figure 1:**
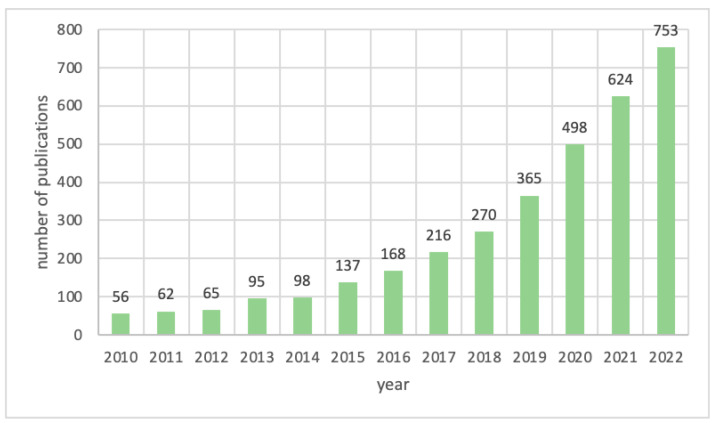
Numbers of publications mentioning PA-derived carbon materials over the last 10 years, according to the ScienceDirect database.

Recently, PA has garnered the attention of materials scientists owing to its potential applications in the construction of sustainable, functional materials for catalytic [37], electrochemical [38,39], and flame-retardant materials [40], as well as its biomolecular sensing capabilities [41]. Figure 1 outlines the last decade’s progress in the development of functional carbon materials with PA.

**Figure 2:**
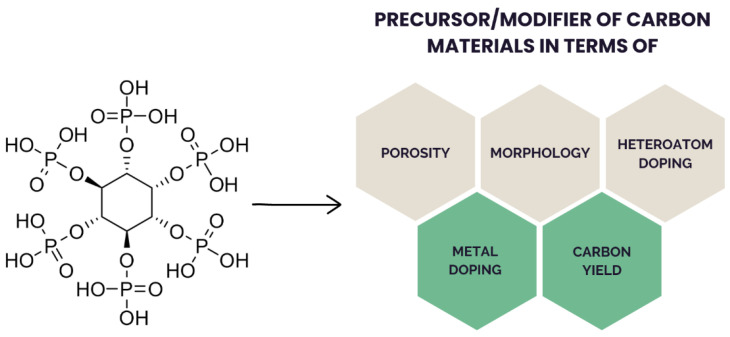
Structure of PA and its possible influence on the derived carbon materials.

PA (Figure 2) is a sugar (inositol) derivative with a structure that is rich in phosphate groups (11% phosphorus; 44% oxygen; 11% carbon), thus making PA an attractive precursor for manufacturing P-doped carbon materials upon carbonization.

At acidic pH, the phosphate groups on PA are fully protonated, providing a unique structure and affecting its physicochemical properties. At neutral pH, PA can form complexes with multivalent metal ions, such as calcium, zinc, or iron [42,43]. Therefore, upon carbonization, it can generate materials simultaneously that are doped with phosphorus and metal (e.g., Fe [20,44], Co [45], Ni [46], Sn [47], Al [48], Mn [49]). The six phosphate groups exhibit strong affinity for amino groups through ionic interactions or hydrogen bonds, enabling PA to serve as a crosslinking agent [50,51]. During pyrolysis, in the temperature range of approximately 500–800 °C, PA can produce carbon materials with acidic properties due to functionalization with phosphonate and phosphate groups [21]. Moreover, the presence of –PO_4_ groups on the surface provides excellent hydrophilicity [52]. Thus, PA can also function as a surfactant during the formation of hydrogels [53].

This review presents the most common synthetic protocols for preparing carbon materials with PA and discusses their influence on the physiochemical and electrochemical properties of the derived materials. The most recent approaches are discussed, covering the state of the art since 2015. We also describe the electrocatalytic activities of biomass-derived/functionalized materials toward the ORR to explore their potential applications in energy conversion and storage.

## 2. Methods for Synthesizing Heteroatom-Doped Carbon Materials

The most common strategies for synthesizing carbon materials using PA involve the direct pyrolysis of PA, which acts as both the carbon source and the heteroatom source [5,20,53,54]. The addition of a hard template (often silica) into the precursor mixture provides additional porosity when it is removed after the pyrolysis step [20,49,52,55,56]. Another strategy involves the chemical or thermal modification of previously prepared carbon nanomaterials with PA; in this case, PA functions as an activator (improving the porosity/microstructure) and/or heteroatom doping agent [57,58,59]. The properties of the as-prepared materials (e.g., structure, composition, catalytic performance) depend heavily on the precursor (or mixture of precursors), carbonization conditions, and template/support (if used).

### 2.1. Template-Free Synthesis

Although template-assisted synthetic methods can yield carbon materials with high surface area and high pore volume, they also require expensive templates and several synthetic steps, rendering them costly and time-consuming [60,61,62]. Thus, template-free synthetic methods are more attractive, especially for the large-scale synthesis of electrocatalysts (Table 1).

Jintao Zhang et al. synthesized materials by carbonizing hydrogels obtained following the polymerization of aniline in the presence of PA [53]. The first step involved the formation of a PA-based protic salt through a simple reaction between PA and amine (aniline), followed by polymerization of aniline in the presence of NH_4_S_2_O_8_. Next, the obtained hydrogel was freeze-dried to produce an aerogel, which was subsequently carbonized. The microstructure of the resulting carbon material depended on the molar ratio of aniline to phytic acid. At a relatively low concentration of aniline (aniline:phytic acid 1:1), the polymerization process generated spherical micelles, which later transformed into larger spheres and, upon carbonization, yielded aggregated coralliform structures. However, as the concentration of aniline increased, the spherical micelles transformed into cylindrical structures, leading to the formation of a hierarchical porous carbon material composed of interconnected fibers. Similar synthetic approaches based on hydrogel carbonization have been applied to fabricate chitosan-derived carbon materials [51].

In another approach, *o*-phenylenediamine was polymerized in the presence of an oxidant to produce hollow nanospheres, which were further treated with an aqueous solution of PA [63]. The subsequently dried materials were subjected to carbonization at 900 °C. The amphiphilic nature of the *o*-phenylenediamine monomers allowed them to form droplets in water without introducing an additional surfactant. When monomer polymerization occurred on the droplet surfaces, hollow structures were generated with additional holes on the surface. This method is often thought of as self-templating. The procedure can be modified by simultaneously mixing *o*-phenylenediamine, PA, and iron(III) chloride to produce P-, Fe-, and N-doped carbon materials [64]. The distribution and content of N, P, and Fe atoms in the obtained material is precisely controlled by the acid–base reaction between the PA and *o*-phenylenediamine, as well as the coordination between the remaining PA hydroxyl groups and iron ions.

**Table 1:** Physicochemical properties of PA-derived carbon materials obtained via template-free synthesis.

Electrocatalyst	Precursors	Doped Atoms (wt%)	Surface Area (m^2^ g^−1^)	Porous Structure	Ref.
Template-Free Synthesis					
NPCNS_700T	Phytic acid, chitosan	N: 6.40 P: 5.80	-	-	[54]
N,P-HLC	Phytic acid, melamine, glucose	N: 4.92 P: 0.55	422	-	[65]
FeP@SA-Fe/HC	Phytic acid, melamine, iron nitrate, 2-aminoterephthalic acid	N: 3.17 P: 4.55 Fe: 0.45	111	Average pore size: 3.87 nm	[66]
NPFe-C	Phytic acid, melamine, iron(III) chloride hexahydrate	N: 3.12 P: 3.51 Fe: 0.81	775	Micropore distribution: 0.90 nm Mesopore distribution: 2–35 nm	[67]
NP+NG/PG	Phytic acid, 2,6-diaminopyridine, 5-aminouracil	N: 4.52 P: 0.67	1114	Micropore distribution: 1–2 nm Mesopore distribution: 2–10 nm	[68]
N, P, O-Carbon-PA	Phytic acid, o-phenylenediamine, ammonium hydroxide	N: 5.52 P: 2.15	367	Average pore size: 4.00 nm	[63]
P-Fe-NC	Phytic acid, o-phenylenediamine, ferric chloride hexahydrate	N: 3.08 P: 1.15 Fe: 0.42	1216	Average pore size: 1.87 nm	[64]
Fe, P, N-Carbon	Phytic acid, o-phenylenediamine, iron(III) nitrate nonahydrate	N: 4.98 P: 2.97 Fe: 1.68	458	Average pore size: 4.00 nm Pore volume: 0.38 cm^3^ g^−1^	[69]
Fe−P−C	Phytic acid, iron chloride	P: 3.10 Fe: 0.50	1371	Pore size distribution: >3.00 nm	[44]
FeP/C	Phytic acid, iron(III) nitrate nonahydrate	P: 15.00 Fe: 7.00	1269	Average pore size: 0.67 nm	[70]
PANI-Fe/PA-N1050	Phytic acid, aniline, ferric chloride hexahydrate	N: 2.67 P: 1.11 Fe: 0.42	-	-	[71]
FeP@NPCs	Phytic acid, folic acid, iron chloride	N: 10.09 P: 1.24 Fe: 0.64	381	Average pore size: 2.10 nm	[6]
FCPA-900	Phytic acid, ferric chloride hexahydrate, cobalt chloride hexahydrate	P: 0.90 Fe: 0.16 Co: 1.20	1646	Micropore size distribution: 1 nm Mesopore size distribution: 5–25 nm	[45]
Co_2_P_2_O_7_/C@ N,P−C HNTs	Phytic acid, aniline, urea, cobalt nitrate hexahydrate	-	459	Average pore size: 2.63 nm	[72]
NPS-PC	Phytic acid, zinc pyrithione	N: 3.74 P: 6.61 S: 0.92	712	Average pore size: 3.40 nm Pore volume 0.40 cm^3^ g^−1^	[73]
Fe_2_P/FeP-PNC	Phytic acid, urea, glucose, ferric chloride	N: 10.25 P: 0.97 Fe: 0.73	-	-	[74]
NPMC-1100	Phytic acid, aniline	N: 1.80 P: 0.10	1663	Pore size distribution >10.00 nm Pore volume: 0.42 cm^3^ g^−1^	[53]
NPCNFs	Phytic acid, aniline	N: 8.00 P: 0.90	741	Average pore size: 14.42 nm Pore volume: 0.45 cm^3^ g^−1^	[75]
Fe-N/P/C-850	Phytic acid, aniline, ferrocene	N: 3.65 P: 3.64 Fe: 0.87	615	-	[76]
PNC	Phytic acid, 2,6-diamino pyridine	N: 1.92 P: 1.65	952	Average pore size: ~4.00 nm	[77]
NPMC-1000	Phytic acid, glucose, urea	N: 5.00 P: 2.33	1026	Average pore size: ~0.90 nm Pore volume: 1.12 cm^3^ g^−1^	[78]
NiCoP/NSP-HPCNS	Phytic acid, thiourea, cobalt acetate, nickel phthalocyanine	N: 6.00 P: 1.50 S: 0.32 Ni: 0.79 Co: 0.75	32	Average pore size: 3.97 nm	[79]
NPMC/CoFe	Phytic acid, dicyandiamide, iron(III) nitrate nonahydrate, cobalt nitrate hexahydrate	N: 4.82 P: 1.37 Fe: 12.51 Co: 5.68	679	Average pore size: 19.65 nm	[80]
CoP NPs/CNSs	Phytic acid, melamine, cobalt(II) acetate tetrahydrate	-	234	Average pore size: 2.00 nm Pore volume: 0.32 cm^3^ g^−1^	[81]
Fe_2_P/NPCs	Phytic acid, aniline, trimethylbenzene, citric acid, polyethylene-polypropylene-glycol, ferric chloride	N: 2.68 P: 2.18 Fe: 0.91	523	Average pore size: ~3.46 nm	[82]
NPC1000	Phytic acid, gelatin powder	N: 3.60 P: 2.07	1056	Average pore size: 3.37 nm	[83]

### 2.2. Template-Assisted Synthesis

To increase the porosity of carbon materials, templating methods can be applied to facilitate the formation of an ordered microstructure and a fine-tuned architecture. In this approach, a template assists the formation of pores with narrow size distribution, thereby increasing the surface area, mass transport, and diffusion, which are essential for catalytic applications [84] (Table 2). Duraisamy et al. synthesized homogeneous N- and P-doped carbon spheres through a polymerization reaction [55]. In the first synthetic step, tetraethoxysilane (TEOS) was used to prepare silica spheres, which were subsequently coated with polydopamine and PA. The as-prepared hybrids were carbonized at 900 °C, and then the silica was removed to produce N- and P-doped hollow mesoporous spheres.

Zhou et. al. used TEOS to modify N- and P-doped graphene sheets obtained by polymerizing aniline in the presence of PA on a graphene oxide surface [57]. This approach combined post-functionalization and hard templating methods, preventing the restacking of graphene sheets and creating macropores to facilitate reagent diffusion.

Commercially available silica nanoparticles are also often used as a hard template. Zhu et al. fabricated N- and P-doped mesoporous carbon composites, which were also coordinated with manganese atoms [49]. They employed a facile synthesis strategy that involved mixing manganese nitrate, *o*-phenylenediamine, and a silica colloid in a PA solution, followed by carbonization at 900 °C. Then, alkaline and acidic leaching of the as-received carbon residue removed the matrix and the unreactive Mn species. The resulting materials had highly porous structures with uniform pores (~10 nm) resulting from the applied template. The material had a surface area of 891 m^2^ g^−1^, indicating that the hard template may not necessarily lead to a higher surface area but, rather, may cause a change in morphology. For comparison, the P-, Fe-, and N-doped carbon material described above (Section 2.1) attained a specific surface area (S_BET_) as high as 1216 m^2^ g^−1^ without using a template [64]. The superior pore-generating ability of PA is discussed further in Section 3.

Although silica is the most commonly used hard template, other materials—such as inorganic salts or oxides (e.g., NaCl, KCl, MgO, CaCO_3_) [85], carbon spheres [86], and organic polymer spheres [87,88]—can also effectively play this role. One interesting example of a hard template is rubidium chloride [52], which can be mixed with PA to afford aggregated, spherical carbon particles (<30 μm) after annealing at 800 °C. The addition of an alkaline metal, which could be easily removed from the obtained catalyst after a thermal acid treatment, increased the S_BET_ from 793 to 1380 m^2^ g^−1^.

### 2.3. Post-Modification

PA can be used as a modifier of various carbon (nano)materials—including graphene oxide (GO) [89], active carbon [90], graphite carbon nitride [22,74], manganese [91] or cobalt [72] oxide nanorods, carbon nanotubes (CNTs) [92], carbon nanospheres [56], cellulose carbon nanofibers [93], metal-organic frameworks [94], and cerium oxide nanosheets [95]—to tune their properties and enhance their electrochemical activities (Table 3).

Jang et al. modified GO by mixing it with PA and refluxing the mixture for 12 h. Continued stirring at 90 °C for an additional 12 h with an aqueous ammonium hydroxide solution led to the precipitation of agglomerated, black N- and P-doped particles [96]. The powder obtained using this straightforward low-temperature procedure had a specific surface area of 354 m^2^ g^−1^ and exhibited electrocatalytic ORR activity.

To inhibit GO nanosheet aggregation, Zhang et al. functionalized GO with poly(oxypropylene) diamine, which was further reacted with PA to produce a supramolecular protic salt [59]. In the first stage, GO and poly(oxypropylene) diamine were dispersed in water, and a homogeneous suspension was obtained following ultrasonication. Afterwards, the supramolecular aggregate was formed in the form of a precipitate, to which PA was slowly added, followed by filtration and drying at 80 °C. Subsequent functionalization with Co- and Mn-layered double hydroxides (LDHs) via a hydrothermal process yielded a highly crosslinked hydrogel. The authors conducted an additional freeze-drying procedure to promote the uniform dispersion of CoMn-LDH nanoplates on N- and P-doped GO composites.

Wang et al. developed an interesting approach where PA was used as a precursor for obtaining carbon dots decorating the surface of GO [97]. The GO surface has oxygen-containing groups and multiple defects, which provide accessible active sites for decoration with phosphate linkages to PA. Upon anchoring, these moieties were further converted into carbon dots under hydrothermal conditions. This strategy enabled the production of a composite comprising 7–9 wt% carbon derived from biomass.

**Figure 3:**
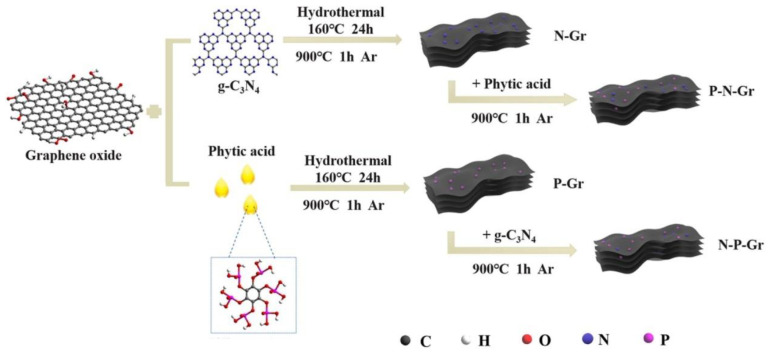
Schematic illustration of different synthesis strategies for N- and P-doped graphene materials. Reproduced with permission from [22]. Copyright 2022 Elsevier Inc. All rights reserved.

Another GO modification method using g-C_3_N_4_ and PA was proposed by Liao et al. [22]. The authors modified GO via two synthetic routes (Figure 3). The first involved treating GO with g-C_3_N_4_ under hydrothermal conditions, followed by pyrolysis with PA at 900 °C. The second method used the reversed order, i.e., GO was treated with PA under hydrothermal conditions, followed by pyrolysis with g-C_3_N_4_. The order of the steps influenced the specific surface area and the extent of GO doping. Initial treatment with a N source led to a material with high S_BET_ (935 m^2^ g^−1^), higher P doping (2.6%), and higher O doping (13.6%), whereas initial treatment with PA produced a material with lower S_BET_ (125 m^2^ g^−1^), P doping (1.2%), and O doping (7.7%).

GO can also be fine-tuned with PA by exclusively using high-temperature treatments. Li et al. polymerized aniline in the presence of PA on the surface of GO [98]. The hydroxyl and carboxyl groups on the GO surface enabled the formation of a conformal coating through crosslinks formed between GO, aniline, and PA during polymerization. Following thermal treatment at 850 °C, the material adopted a sandwich-like hierarchically porous structure. Similar structures were observed when using melamine instead of aniline, without a polymerization step [58].

Razmjooei et al. functionalized PA with iron(II) chloride to produce ferric phytate, which was then used for the modification of GO. Introducing Fe atoms into the composite before carbonization increased the degree of P doping and enhanced the electrochemical activity of the obtained composite [99].

**Table 3:** Physicochemical properties of PA-derived carbon materials obtained by post-modification.

Electrocatalyst	Precursors	Doped Atoms (wt%)	Surface Area (m^2^ g^−1^)	Porous Structure	Lit.
Synthesis by Post-Modification					
N,P-MC	Phytic acid, pyrrole, polystyrene microsphere	N: 3.56 P: 0.60	305	Micropore average size: 0.9 nm Mesopore average size: 10 nm	[100]
N,P-Fe/C	Phytic acid, aniline, polystyrene microspheres, iron acetylacetonate	N: 2.64 P: 1.65 Fe: 0.81	460	Average pore size: 5.02 nm	[101]
N,P-HPC	Phytic acid, dicyandiamide, cattle-bone-derived carbon	N: 3.20 P: 3.96	1516	Micropore size distribution: 0.8–1.1 nm Mesopore size distribution: 2.8–4.8 nm	[5]
S,N,P-HPC	Phytic acid, thiourea, dicyandiamide, cattle bone derived carbon	N: 4.35 P: 2.96 S: 1.29	1533	Average pore size: 1.40 nm	[102]
MPSA/GO	Phytic acid, melamine, graphene oxide	N: 3.20 P: 2.10	375	Average pore size: 2.50 nm	[58]
NPC/G	Phytic acid, chitosan, graphene oxide	N: 0.93 P: 0.65	1824	Average pore size: 2.10 nm	[51]
NP8-VACNT-GF	Phytic acid, aniline, cobalt acetate, iron chloride, graphene foam	N: 9.40 P: 0.70 Fe: 0.20 Co: 0.10 Cu: 1.60	-	Pore size distribution: 5−10 nm	[60]
P-N-Gr	Phytic acid, graphene oxide, graphitic carbon nitride	N: 0.73 P: 2.61	935	Average pore size: 3.71 nm Pore volume: 0.87 cm^3^ g^−1^	[22]
GNP–900	Phytic acid, polyethylenimine, graphene oxide	N: 2.61 P: 2.26	613	Average pore size: 3.48 nm	[103]
CoMn-LDH/NPGA	PA, poly(oxyproylene) diamine, cobalt nitrate hexahydrate, manganese nitrate tetrahydrate, graphene oxide	N: 4.20 P: 3.34 Co: 17.87 Mn: 4.83	106	Pore size distribution: 4.0–30.0 nm	[59]
P-CD/G	Phytic acid, graphene oxide	P: 2.31	448	Average pore size: 3.50 nm	[97]
N,P-GCNS	Phytic acid, aniline, graphene oxide	N: 4.71 P: 1.72	900	-	[98]
N-P-rG-O	Phytic acid, ammonium hydroxide graphite oxide	N: 7.7 P: 0.61	354	Pore size distribution <40 nm	[96]
GPFe	Phytic acid, iron(II) chloride, graphene oxide	P: 0.84 Fe: 0.6	612	Average pore size: 3.17 nm Pore volume: 0.56 cm^3^ g^−1^	[99]
NPS G_2_	Phytic acid, ethylene glycol reduction, graphene oxide	N: 5.57 P: 3.38 S: 0.83	605	Average pore size: 0.55 nm Pore volume: 0.27 cm^3^ g^−1^	[104]
NC@CoPx/PyCNTs	Phytic acid, melamine, 4-aminopyridine, 5,10,15,20-tetra(4-pyridyl)porphyrin, cobalt acetate tetrahydrate, multiwalled carbon nanotubes	N: 7.90 P: 2.26 Co: 5.08	389	Pore size distribution: 2–30 nm	[92]
NPC@AC	Phytic acid, aniline, activated carbon	N: 4.54 P: 2.21	649	Pore size distribution <10.00 nm Pore volume: 0.66 cm^3^ g^−1^	[90]
Co_3_O_4_/NPC	Phytic acid, melamine, urea, ethylene glycol solution, cobalt acetate tetrahydrate, carbon black	N: 1.20 P: 0.09 Co: 2.10	-	-	[105]
NSC/MPA-5	Phytic acid, melamine, ammonium thiocyanate, cellulose nanofibril	N: 3.30 P: 2.50 S: 0.60	682	Pore size distribution: >20.00 nm	[93]
2.5Co_2_P-NPC-CeO_2_	Phytic acid, dopamine CeO_2_ nanosheets, cobalt nitrate hexahydrate	P: 5.12 Ce: 6.49 Co: 1.11	-	-	[95]
MnO_2_@PANI-800	Phytic acid, aniline, manganese(II) sulfate monohydrate	N: 18.91 P: 1.97 Mn: 6.11	-	Diameter: ~130 nm Carbon shell thickness: ~25 nm	[91]
Co_2_P@NPC	Phytic acid, melamine, dimethylimidazole, cobalt nitrate hexahydrate, ZIF-67	Co: 40.2	259	Average size: ~18.2 nm	[94]
PA-ZIF-67–900	Phytic acid, ZIF-67	-	292	Pore size distribution: 1.74–32.00 nm	[106]
CMD-900-4	Phytic acid, diaminonaphthalene, g-C_3_N_4_		838	Pore size distribution: 6.00–10.00 nm	[107]

**Figure 4:**
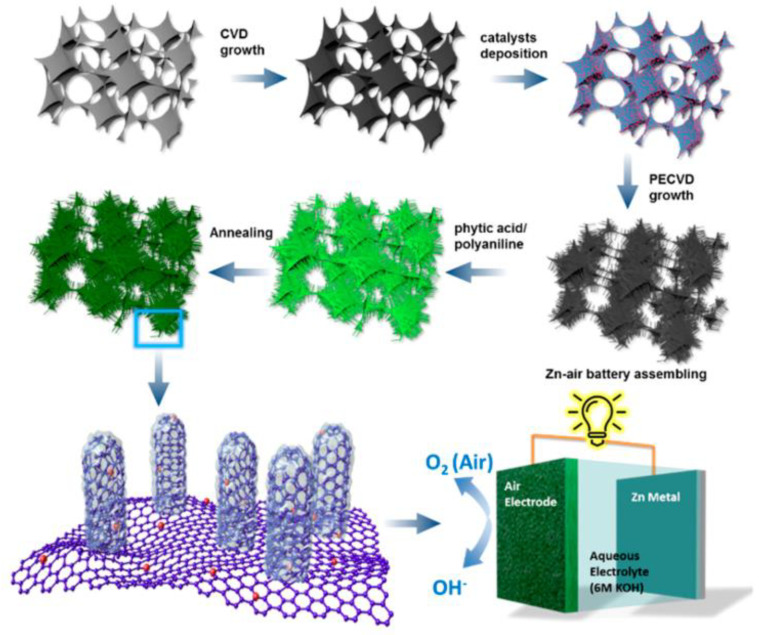
Procedure for synthesizing P- and N-doped vertically aligned carbon nanotubes on graphene foam (NP−VACNTs−GF). Reproduced with permission from [60]. Copyright 2019 American Chemical Society.

Other nanomaterials (e.g., oxides) have also been successfully modified with PA or its salts to generate a carbon layer on the surface, thereby boosting their electrochemical activity. The polymerization of aniline on the surface of MnO_2_ nanorods in the presence of PA yielded a porous shell following carbonization at 800 °C. The core–shell approach produced a material with uniformly distributed Mn, O, N, and P atoms. Additionally, N- and P-doped carbon layers have also been formed on hollow carbon spheres [56] and vertically aligned carbon nanotubes on graphene foam [60]. The latter is an advanced, durable, free-standing structure synthesized via a multistage process (Figure 4). The synthesis procedure involved the formation of a graphene foam, which was covered with a bimetallic Fe−Co catalyst that promoted the growth of CNTs using plasma-enhanced chemical vapor deposition. The surface of the resulting hybrid material was oxidized using HNO_3_; this treatment formed –COOH groups on the surface, which could then anchor the aniline molecules. Polymerization of aniline in the presence of PA created a core–shell structure after annealing at 700–1000 °C. Specifically, the outer layer comprised numerous dopants and defects, which served as catalytic active sites, and the core was composed of highly conductive CNTs.

## 3. Effects of Phytic Acid on the Physicochemical Properties of Doped Carbon Materials

Research has shown that heteroatom doping can change the structure as well as the chemical and electronic properties of carbon materials, promoting electrochemical reactions on their surface [1,84,108]. Biomass-derived PA represents an attractive phosphorus source and a promising alternative to inorganic acids or salts, such as H_3_PO_4_, NaH_2_PO_2_, and NH_4_H_2_PO_4_. Moreover, the porosity and developed surface area of doped carbon materials increase the availability of active centers for reactants [109]. The presence of micro-, meso- and macropores can also facilitate the transport of oxygen and electrolyte ions during the electrochemical reaction by shortening the diffusion pathways [110]. This section discusses the influence of PA on the structural properties of carbon materials, i.e., doping level, specific surface area, and average pore size.

### 3.1. Structure of Carbon Materials

The selected precursors and carbonization conditions (e.g., temperature, time, solvent) influence the structure and physicochemical properties of the fabricated carbon materials.

PA primarily serves as a carbon source [44], although during the carbonization process it also plays the role of a porogen [23,49,65,89] and phosphorus dopant [46,54] owing to its numerous phosphate groups.

PA is typically used in the form of an aqueous solution; however, at temperatures above 100 °C, it hydrolyzes to phosphoric acid (H_3_PO_4_; Equation (1)). Between 400 and 500 °C, H_3_PO_4_ is transformed into phosphorus pentoxide (P_2_O_5_), which creates pores upon sublimation (Equation (2)). When the temperature is further increased to 900 °C, some of the remaining P_2_O_5_ is reduced, thereby producing red phosphorus, which acts as a self-sacrificing template to form mesopores (Equation (3)) [90,91].

C_6_H_18_O_24_P_6_ + 6H_2_O → 6H_3_PO_4_ + C_6_H_12_O_6_ (1)

2H_3_PO_4_ → P_2_O_5_ + 3H_2_O (2)

P_2_O_5_ + 5C → 2P + 5CO (3)

Thus, micro- and mesopores are formed during thermal treatment via dehydration or dephosphorization. These processes produce gases, such as H_2_O, CO, and P_2_O_5_, which create pores when they escape the carbon structure.

Carbonization of PA alone can result in a highly porous carbon structure, where the specific surface area depends on the annealing temperature. Prior to hydrothermal treatment at 120 °C for 24 h, PA carbonized at 500 °C yielded carbon residues with low S_BET_ (34 m^2^ g^−1^). However, increasing the annealing temperature to 600 °C increased the S_BET_ value significantly (to 1039 m^2^ g^−1^). Increasing the annealing temperature to 800 °C further increased the S_BET_ to 1637 m^2^ g^−1^ [21]. Meanwhile, carbon residues with an S_BET_ of only 577 m^2^ g^−1^ were reported for PA carbonized at 900 °C [44].

For carbon materials that are also doped with nitrogen, an increase in the specific surface area may be due to NH_3_ released during the decomposition of the precursor (i.e., nitrogen source). Zan et al. compared P-doped and P/N-co-doped porous carbon materials derived from cattle bones [5]. The material treated with PA had an S_BET_ of 1389 m^2^ g^−1^, whereas the analogous material treated with both PA and dicyandiamide reached an S_BET_ of 1516 m^2^ g^−1^. Additional N and P doping contributed to the development of larger mesopores compared with the unmodified material.

**Figure 5:**
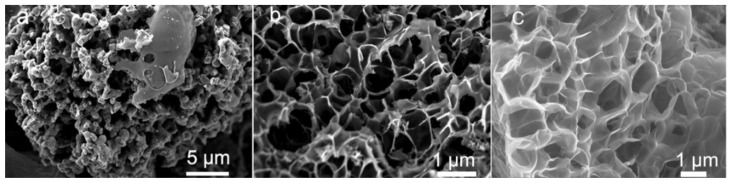
SEM images of synthesized honeycomb-like carbon materials using different amounts of PA: (**a**) 0 mL, (**b**) 1 mL, and (**c**) 5 mL. Reproduced with permission from [65]. Copyright 2021 Elsevier Inc. All rights reserved.

The amount of PA used as a precursor can also significantly influence the morphology of the resulting material. Zhang et al. reported that as the amount of PA precursor increased, the morphology of the investigated honeycomb-like carbon materials became fluffier because the overloaded PA triggered the collapse of the mesopores (Figure 5) [65].

**Figure 6:**
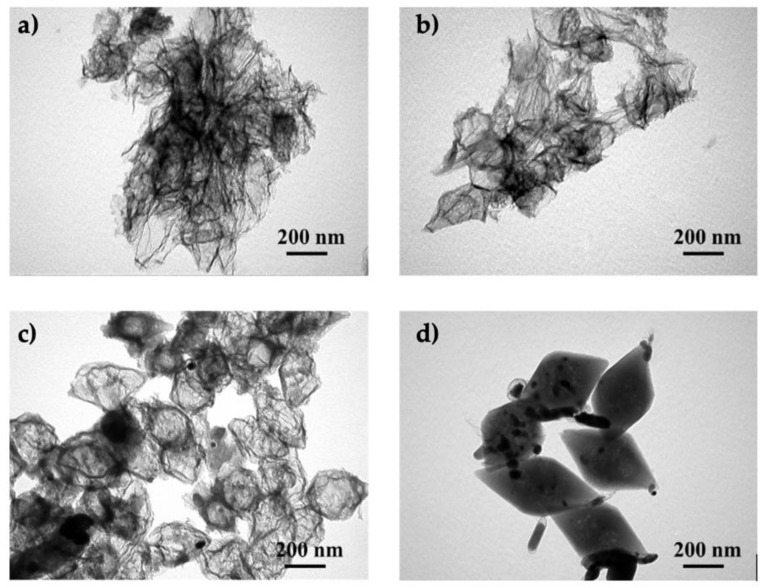
The effects of different PA loadings for the modification of hollow carbon nanostructures: Morphologies of materials treated with (**a**) 1 mL, (**b**) 0.5 mL, (**c**) 0.2 mL, and (**d**) 0.1 mL of PA. Reproduced with permission from [66]. Copyright 2020 Elsevier Inc. All rights reserved.

The amount of PA when used as a modifier can also drastically influence the microstructure of the resulting material. Yang et al. observed that if the PA loading when modifying hollow carbon nanostructures (prepared from an iron metal–organic framework) was too high, their spindle shape was completely destroyed (Figure 6a,b) [66]. Similarly, if the amount of PA was too low, it was difficult to obtain a porous and doped carbon composite. Therefore, selecting the PA loading is often a compromise between achieving a desired structure and a suitable doping level.

PA is commonly used as an activator to increase the specific surface area of a material. The S_BET_ values of carbon aerogels prepared using chitosan and PA as precursors were improved significantly (from 279 to 737 m^2^ g^−1^) when the mass ratio of PA to chitosan was increased from 0.5 to 2 [51]. Moreover, when the aerogel was incorporated into graphene and carbonized at 1000 °C, the composite reached an S_BET_ as high as 1824 m^2^ g^−1^, which is the best result among all of the materials described in this review. Liu et al. confirmed that the presence of PA (acting as a porogen) could significantly increase the surface area and porosity of graphene-based materials [51]. The formation of P_2_O_5_ during carbonization distorted the graphitic structure, forming pores and wrinkles [99]. Moreover, the addition of PA can contribute to increased spacing between graphite layers because of the larger atomic radius of P relative to that of carbon [101].

A recent study demonstrated that the amount of PA and the duration of its addition when preparing functional carbon materials can influence their ultimate shape and size. Duraisamy et al. investigated the loading and duration of the addition of PA during the synthesis of carbon spheres using silica, dopamine hydrochloride (DA), and PA. Adding PA to the DA–SiO_2_ mixture for 10 h resulted in the formation of spherical, joined particles (diameter ~198 nm) [55]. Extending the duration of the addition of PA to 30 h generated spheres with larger diameters (~264 nm) and thicker sphere walls (thickness increased from 25 to 53 nm), owing to a greater degree of aggregation of polydopamine and PA. Interestingly, the shell thickness and sphere diameter decreased as the PA loading increased. The authors explained this phenomenon based on the fact that the change in pH during the reaction potentially hindered the formation of certain structures.

### 3.2. Chemical Doping

The introduction of P atoms (electronegativity = 2.19) into a carbon structure can induce interactions with C atoms (electronegativity = 2.55), thereby triggering the redistribution of charge and spin density [1]. The disrupted electron neutrality of the carbon matrix increases the electrocatalytic ORR activity of doped carbon materials [44]. Different-sized heteroatoms can also disturb the geometry of the carbon lattice and generate structural defects, which can also enhance the material’s catalytic properties.

Obtaining uniform distribution of heteroatoms throughout the carbon network remains a challenge; however, PA (which contains the same number of carbon and phosphorus atoms) may enable uniform doping during the carbonization process [21]. Moreover, owing to its carbon-rich structure, low cost, availability, and bio-derived origin, PA has become a commonly used carbon source, following the trend of synthesizing functional carbon materials using non-toxic and abundant plants, agricultural and forestry waste products, and microorganisms [111,112,113,114]. Direct carbonization of PA produces P-doped carbon materials without an additional carbon source [20,96]. Additionally, pyrolyzing a mixture of PA and iron chloride yielded a nonprecious-metal-based carbon catalyst [44]. Finally, PA has been used to fabricate P-doped carbon coatings on various inorganic materials [72,97].

#### 3.2.1. Doping with Phosphorous and Other Atoms

Although it is not as effective as N doping, P doping can enhance the electrocatalytic properties of carbon materials. Density functional theory (DFT) calculations on P-doped graphene suggest that the phosphorus dopant serves as the active site to adsorb oxygen species during ORR, because of the positive charge of phosphorus atoms (0.652 a.u.) compared with the more negative charge of the neighboring carbon atoms (0.298 a.u.) [115,116]. The calculations demonstrated that P-doped graphene exhibits higher charge mobility and better donor–acceptor properties than analogous unmodified materials. There are several configurations whereby phosphorus atoms can integrate into the carbon structure. X-ray photoelectron spectroscopy (XPS) analysis of PA carbonized at 600 °C revealed two types of phosphorus species: including P–C and P–O groups, observed at ~132 and 134 eV, respectively [20,99,117]. The P–O groups can be a part of phosphonate, phosphate, and/or phosphine oxide groups, which build up around the edge of the carbon lattice. The phosphonate and phosphate groups endow the carbon material with additional Brønsted acidity [21].

Upon increasing the pyrolysis temperature, the overall content of phosphorus incorporated into the carbon structure decreases (similar phenomena have been observed with other heteroatoms). Direct carbonization of PA at 500 °C yielded carbon residues with P content up to 13.59%, which decreased to 3.6% after raising the temperature to 800 °C [18]. In general, high temperatures promote the conversion of phosphorous-containing functional groups into atomic phosphorus in the carbon network [50,55,57].

Co-doping in P-doped carbon materials is typically accomplished with N doping, which can lead to increased ORR activity owing to the synergistic electronic action of both dopants (e.g., P doping makes the N sites more catalytically active) [118]. The highest phosphorus content reported for P- and N-doped carbon synthesized at 600 °C was 6.9%; this material was derived from chitosan and had a nitrogen content of 6.1% [54]. However, an excessive amount of PA promotes the loss of nitrogen-containing species from the carbon structure, because the higher amount of gases produced during pyrolysis may facilitate their escape [22].

#### 3.2.2. Doping with Phosphorus and Nonprecious Metals

Owing to its strong complexing capabilities, PA can facilitate co-doping with metals, while also promoting their uniform distribution [70], limiting agglomeration [23], increasing the carbonization efficiency [74], and improving the resulting material’s mechanical strength [70].

Deng et al. developed N/P/Fe-tri-doped carbon foams using a simple PA-assisted self-templating strategy and showed that the acid–base reaction between PA and *o*-phenylenediamine enabled control over the contents of N and P atoms; meanwhile, the contents of nonprecious metals could be controlled by coordinating Fe to the hydroxyl groups, leading to the uniform distribution of all dopants [64]. Xue at al. demonstrated that carbon nitride (g-C_3_N_4_) sheets modified with PA and Fe were uniformly covered with dopant; however, the analogous material prepared without PA had a homogeneous distribution of C and N, but suffered from Fe aggregation. The authors observed that the Fe aggregates catalyzed the combustion of g-C_3_N_4_, thus decreasing the efficiency of the synthetic process [74].

**Figure 7:**
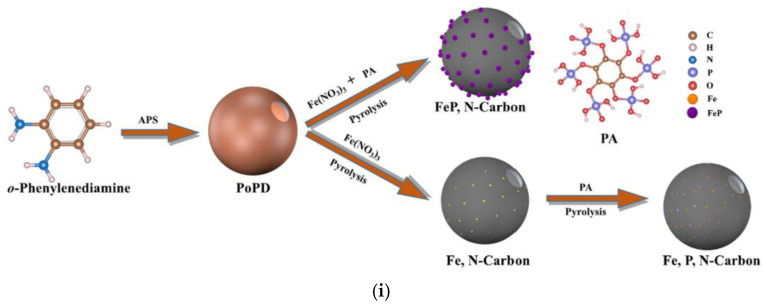

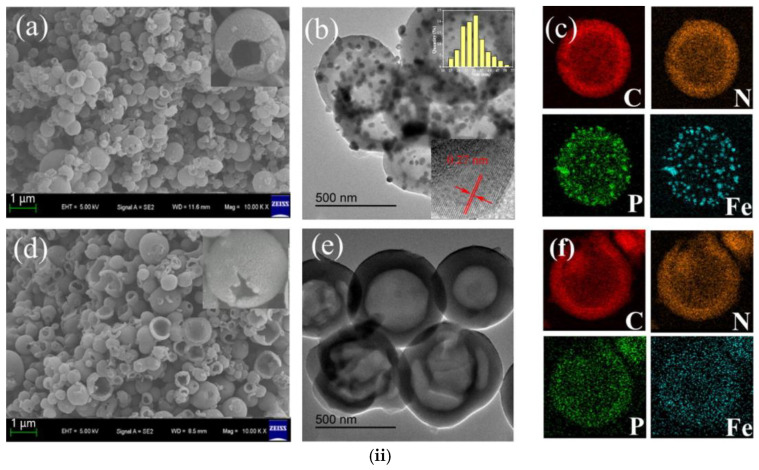
(**i**) Schematic diagram illustrating the synthesis of tri-doped hollow nanospheres (carbon atoms–brown, hydrogen atoms–white, nitrogen atoms–blue, phosphorous atoms- ?, oxygen atoms–red, iron atoms–orange, iron-phosphorous nanoclusters–purple). (**ii**) (**a**) Scanning electron microscopy (SEM), (**b**) transmission electron microscopy (TEM), and (**c**) energy-dispersive X-ray spectroscopy (EDS) images of the materials synthesized via simultaneous addition of precursors; (**d**) SEM, (**e**) TEM, and (**f**) EDS images of the material synthesized via the two-step procedure. Reproduced with permission from [69]. Copyright 2021 Elsevier Inc. All rights reserved.

Recently, Li et al. showed that the order of precursor addition during the synthesis of tri-doped carbon materials is relevant [69]. The simultaneous addition of iron precursors and PA to hollow nanospheres obtained via polymerization of *o*-phenylenediamine led to agglomeration of iron after carbonization (Figure 7). However, when double carbonization was performed (after adding Fe precursor, and again after adding PA), uniform doping was achieved.

Wang et al. elucidated the phase structure of Fe_x_P in N- and P-doped nanospheres [82]. They demonstrated that the phase structure of Fe_x_P depends on the Fe/P molar ratio used in the hydrothermal synthesis. Notably, when the Fe/P ratio was small (Fe/P < 0.1), FeP was the main product. However, as the Fe/P ratio increased, Fe_2_P (Fe/P ≈ 0.2) or Fe_3_P (Fe/P > 0.4) dominated. The increased proportion of the iron precursor may induce the formation of smaller nanospheres on the carbon surface during high-temperature treatment [119].

Although carbon materials co-doped with Fe as a nonprecious metal are the most common, Wang et al. studied doped carbon structures prepared using zinc pyrithione and PA as precursors (i.e., Zn/N/S/P-doped) [73]. The incorporation of zinc in the final material only reached 0.37% when PA was added. In contrast, the material obtained without PA contained 2.91% Zn. Therefore, the authors speculated that PA promoted the evaporation of Zn, which acted as an additional pore-shaping agent during this process.

## 4. Oxygen Reduction Performance of PA-Derived Electrocatalysts

PA contributes to carbon materials in terms of doping, introducing defects, and generating porous structures, all of which influence their electrocatalytic ORR performance. Recent progress in the engineering of metal-free electrocatalysts using PA has enabled the development of materials with activities comparable to those of precious metals.

The electrochemical behaviors of newly developed ORR catalysts are commonly screened using a rotating disk electrode (RDE) or a rotating ring-disk electrode (RRDE), which provide data for the Koutecky–Levich analysis. For electrochemical investigations, both methods use carbon powders, typically dispersed in an alcohol–water mixture, which is then deposited on glassy carbon electrodes to form films. The electrochemical performances of recently reported PA-derived carbon materials are summarized in Table 4.

Although nitrogen is the most commonly used element for doping of carbon materials, P doping (effectively implemented by using PA) can increase electrocatalytic ORR performance. Zhang et al. showed that a mesoporous carbon foam co-doped with nitrogen and phosphorus exhibited higher electrocatalytic activity than the analogous material doped exclusively with nitrogen atoms [53]. The N- and P-doped carbon material synthesized at 1000 °C had an onset potential of 0.94 V versus the reversible hydrogen electrode (RHE), i.e., it was comparable to that of Pt/C. Li et al. prepared hollow spheres using various phosphorus sources (e.g., NaH_2_PO_2_, H_3_PO_4_, and PA) and observed the highest electrocatalytic performance (onset potential = 0.85 V) for the PA-modified material [63]. The incorporation of PA led to carbon spheres with the highest phosphorous doping among the studied phosphorus sources. Simultaneous N/P/S doping of carbon materials enhanced their electrocatalytic ORR performance. The tri-doped porous carbon nanosheets with 3.74% N, 6.61% P, 0.92% S, and surface area = 711.6 m^2^ g^−1^ had a more positive half-wave potential (0.91 V) than commercial Pt/C (0.87 V); moreover, they had better stability in alkaline electrolytes and excellent activity in acidic electrolytes [73].

The ultimate content of dopant is also related to the pyrolysis temperature, which simultaneously affects the degree of graphitization. Zhang et al. observed that increasing the pyrolysis temperature from 900 to 1000 °C had a positive influence on the performance of the electrocatalyst because of the higher degree of graphitization, which promoted electrical conductivity [53]. However, further increasing the pyrolysis temperature to 1100 °C led to the decomposition of the dopants and a negative shift of the potential peak.

The electrocatalytic performance of doped carbon materials is also highly dependent on the porosity of the structure. The mesoporous carbon foams developed by Zhang et al. achieved comparable ORR activity relative to that of Pt/C and had a specific surface area as high as 1548 m^2^ g^−1^ [53]. The micropores provide additional surface-active sites, thereby increasing the ORR activity, whereas larger meso- and macropores facilitate reagent transport [120]. As discussed earlier in this review, PA can endow nanomaterials with additional micro- and mesopores, contributing to their increased ORR activity. In addition to controlled doping with various heteroatoms, oxygen functionalities can also enhance the electrochemical activity of carbon materials by increasing the hydrophilicity of the catalyst surface [120]. Accordingly, the oxygen-rich structure of PA can be beneficial for preparing carbon materials with oxygen-containing functional groups.

**Table 4:** ORR performance of PA-derived electrocatalysts.

Electrocatalyst	Catalysts’ Loading (mg cm^−2^)	Electrolyte (mol/dm^3^)	Onset Potential (V vs. RHE)	Half-Wave Potential (V vs. RHE)	Current Density (mA cm^−2^) (V vs. RHE)	Lit.
N,P-HCS	0.46	0.1 KOH	0.88	0.81	5.62	[56]
MnNPC-900	0.25	0.1 KOH	0.95	0.82	5.0	[49]
NPHS-0.4	0.20	0.1 KOH	0.97	0.79	4.7	[55]
FeNPC	0.25	0.1 KOH	1.03	0.88	6.5	[23]
PON/C-“Rb”	0.21	0.1 KOH	1.00	0.87	-	[52]
NPCNS_700T	0.10	0.1 KOH	0.73	-		[54]
FeP@SA-Fe/HC	-	0.1 KOH	0.94	0.84	-	[66]
NP+NG/PG	0.60	0.1 KOH	1.01	0.89	-	[68]
N, P, O-Carbon-PA	0.20	0.1 KOH	0.98	0.84	3.96	[63]
P-Fe-NC	0.50	0.1 KOH	-	0.93	-	[64]
Fe, P, N-Carbon	-	0.1 KOH	1.03	0.90	5.82	[69]
Fe−P−C	-	0.1 KOH	0.95	-	5.01	[44]
	-	0.1 HClO_4_	0.84	-	5.9	
FeP/C	0.20	0.1 KOH	0.86	0.74	-	[70]
PANI-Fe/PA-N1050	-	0.1 NaOH	-	0.84	4.4	[71]
FeP@NPCs	0.20	0.1 KOH	0.94	0.79	5.85	[6]
Co_2_P_2_O_7_/C@ N,P−CHNTs	-	0.1 KOH	-	0.84	4.58	[72]
NPS-PC	-	0.1 KOH	1.06	0.91	4.00	[73]
Fe_2_P/FeP-PNC	0.30	0.1 M KOH	-	0.85	5.54	[74]
	0.30	0.1 HClO_4_	-	0.70	5.31	
NPMC-1100	0.50	0.1 KOH	0.94	0.85	2.00	[53]
PNC	0.40	0.1 HClO_4_	0.91	0.79	-	[77]
NPMC-1000	0.20	0.1 KOH	0.94	0.84	-	[78]
NiCoP/NSP-HPCNS	0.40	0.1 KOH	0.92	0.84	6.00	[79]
NPMC/CoFe	0.40	0.1 KOH	0.98	0.90	5.70	[80]
CoP NPs/CNSs	0.25	0.1 KOH	0.92	0.88	5.4	[81]
Fe2P/NPCs	0.50	0.1 KOH	0.95	0.820	5.58	[82]
NPC1000	0.40	0.1 KOH	0.87	0.78	4.51	[83]
N,P-MC	0.20	0.1 KOH	-	0.84	-	[100]
N,P-Fe/C	0.30	0.1 KOH	0.97	0.89	5.30	[101]
Co_2_P@am-FePO_4_	-	0.1 KOH	1.01	0.91	6.56	[121]
N,P-HPC	0.80	0.1 KOH	-	0.85	-	[5]
S,N,P-HPC-1	0.80	0.1 KOH	-	0.88	-	[102]
NPC/G	0.25	0.1 KOH	0.95	0.81	5.8	[51]
GNP–900	0.28	0.1 KOH	0.96	0.824	-	[103]
CoMn-LDH/NPGA	0.26	0.1 KOH	0.97	0.87	-	[59]
N,P-GCNS	0.14	0.1 KOH	1.01	0.67	5.56	[98]
N-P-rG-O	-	0.1 KOH	0.89	0.69	5.41	[96]
NPS G_2_	-	0.1 KOH	1.09	0.64	4.17	[104]
NC@CoPx/PyCNTs	-	0.1 KOH	0.92	0.80	4.18	[92]
NSC/MPA-5	0.25	0.1 KOH	0.23	0.76	3.3	[93]
2.5Co_2_P-NPC-CeO_2_	-	0.1 KOH	0.88	0.83	5.24	[95]
MnO_2_@PANI-800	0.10	0.1 KOH	0.92	0.76	4.64	[91]
Co_2_P@NPC	0.28	0.1 KOH	0.83	0.77	-	[94]
CMD-900-4	-	0.1 KOH	0.93	0.85	5.86	[107]
FCPA-900	0.20	0.1 KOH	0.87	0.76	5.68	[45]
N,P-HLC	0.15	0.1 KOH	1.0	0.85	6.23	[65]
		0.5 M H_2_SO_4_	0.87	0.67	7.11	
PA-ZIF-67–900	0.42	0.1 KOH	-	0.85	5.00	[106]
P-N-Gr	0.19	0.1 KOH	1.01	0.82	5.98	[22]
Fe-N/P/C-850	-	0.1 KOH	1.05	~0.86	4.50	[76]

Excellent electrocatalytic ORR activities were achieved by incorporating nonprecious metals—such as Fe—into the carbon structure with the support of PA [20,98,104]. The uniformly Fe/P/N-tri-doped carbon material with a specific surface area of 458 m^2^ g^−1^ had a more positive reduction onset potential (1.03 vs. RHE) than commercial Pt/C catalysts and their P- and N-doped analogues [69]. Additionally, Fe- and P-doped GO showed increased ORR performance compared with non-modified GO [51].

## 5. Summary and Outlook

PA represents an excellent precursor for preparing functional carbon materials for electrocatalytic oxygen reduction applications because of its pore-generating and P-doping capabilities, as well as its sustainable character. The synthetic strategies presented herein demonstrate that carbon materials with different shapes, structures, morphological features, defects, and heteroatom doping can be developed with the support of PA. Most importantly, the natural origins of PA make it an attractive alternative to conventional phosphorous precursors, such as inorganic acids or salts. Converting abundant and renewable biomass into functional nanomaterials with high added value via simple and energy-efficient synthetic strategies is a key pillar of green chemistry. Despite the natural origin of PA and its relatively simple production by extraction with aqueous acids, the synthesis of the discussed N- and P-doped carbon materials often involves toxic nitrogen precursors such as *o*-phenylenediamine or 2,6-diaminopyridine. Therefore, to produce truly sustainable carbon materials, all of the precursors and synthesis strategies should be carefully selected. One option could be the replacement of fossil-fuel-derived amines with amino acids (such as waste proteins from agriculture and forestry). Another issue is the high CO_2_ footprint related to the conventional synthesis of carbons (conversion to a hydrogen economy contributes to reducing CO_2_ emissions, while large amounts of CO_2_ are still produced during the synthesis of carbon electrodes). Therefore, alternative environmentally benign synthesis strategies that involve molten salt CO_2_ capture and electrochemical transformation into various carbon products have been intensively investigated [122,123,124,125,126].

Applying rationally designed synthesis strategies using PA can aid in the development of carbon materials with more active sites, thereby enabling electrocatalytic performance comparable or even superior to those of noble metal catalysts. Therefore, understanding and controlling the mechanisms and influence of PA in hydrothermal and high-temperature carbon manufacturing processes can lead to more sustainable carbon materials that can be developed on a large scale. Evolution of proton-exchange membrane fuel cells (PEMFCs) and rechargeable metal–air batteries with electrodes based on fully sustainable carbon materials can contribute to carbon neutrality.

## Figures and Tables

**Table 2:** Physicochemical properties of PA-derived carbon materials obtained via template-assisted synthesis.

Electrocatalyst	Precursors	Doped Atoms (wt%)	Surface Area (m^2^ g^−1^)	Porous Structure	Lit.
Template-Assisted Synthesis					
N,P-HCS-20	Phytic acid, melamine, tetraethyl orthosilicate	N: 3.08 P: 0.64	721	Average pore size: 3.82 nm Pore volume: 2.8 cm^3^ g^−1^	[56]
MnNPC-900	Phytic acid, o-phenylenediamine, silica solution, manganese nitrate	N: 1.59 P: 1.42 Mn: 0.39	891	Average pore size: 4.26 nm	[49]
NPHS-0.4	Phytic acid, dopamine hydrochloride, tetraethyl orthosilicate	N: 2.66 P: 0.21	1120	Average pore size: 7.00 nm Pore volume: 0.36 cm^3^ g^−1^	[55]
NPHG	Phytic acid, aniline, graphene oxide, tetraethyl orthosilicate	N: 8.88 P: 1.62	332	Pore volume: 2.02 cm^3^ g^−1^	[57]
FeNPC	Phytic acid, dopamine hydrochloride, ferric chloride, tetraethyl orthosilicate	N: 2.43 P: 0.04 Fe: 0.61	1656	Average pore size: 5.86 nm Macropore average size: ~120 nm Mesopore average size: ~10 nm	[23]
PON/C-“Rb”	Phytic acid, rubidium chloride	N: 8.41 P: 2.41	1380	-	[52]

## Data Availability

Not applicable.
